# Reintroducing mothur: 10 Years Later

**DOI:** 10.1128/AEM.02343-19

**Published:** 2020-01-07

**Authors:** Patrick D. Schloss

**Affiliations:** a Department of Microbiology and Immunology, University of Michigan, Ann Arbor, Michigan, USA; University of Manchester

**Keywords:** 16S rRNA gene, amplicon, bioinformatics, data analysis, microbial ecology, microbiome

## Abstract

More than 10 years ago, we published the paper describing the mothur software package in *Applied and Environmental Microbiology*. Our goal was to create a comprehensive package that allowed users to analyze amplicon sequence data using the most robust methods available. mothur has helped lead the community through the ongoing sequencing revolution and continues to provide this service to the microbial ecology community. Beyond its success and impact on the field, mothur’s development exposed a series of observations that are generally translatable across science. Perhaps the observation that stands out the most is that all science is done in the context of prevailing ideas and available technologies. Although it is easy to criticize choices that were made 10 years ago through a modern lens, if we were to wait for all of the possible limitations to be solved before proceeding, science would stall. Even preceding the development of mothur, it was necessary to address the most important problems and work backwards to other problems that limited access to robust sequence analysis tools. At the same time, we strive to expand mothur’s capabilities in a data-driven manner to incorporate new ideas and accommodate changes in data and desires of the research community. It has been edifying to see the benefit that a simple set of tools can bring to so many other researchers.

## Abstract (precis)

More than 10 years ago, we published the paper describing the mothur software package in *Applied and Environmental Microbiology*. Our goal was to create a comprehensive package that allowed users to analyze amplicon sequence data using the most robust methods available. mothur has helped lead the community through the ongoing sequencing revolution and continues to provide this service to the microbial ecology community.

## INTRODUCTION

**FIG 1:**
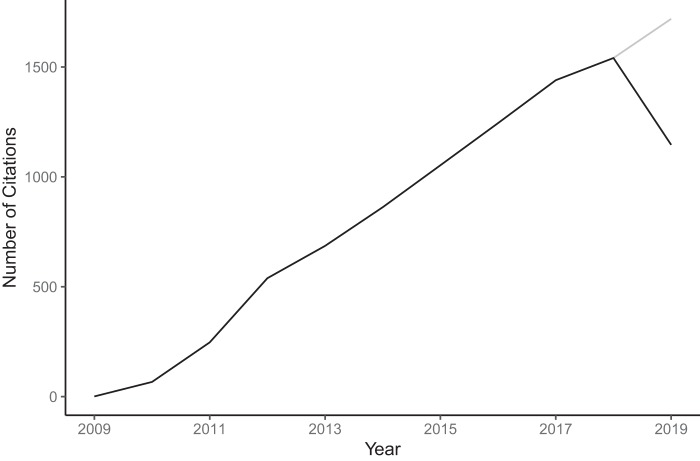
mothur has consistently been a popular software package over the past 10 years with more than 8,800 citations. Citation data taken from the Web of Science (https://www.webofscience.com) on 1 October 2019. The gray line segment depicts the projected number of citations for 2019 based on the current number of citations for the year and historical trends.

Looking back on scientific journeys can be instructive to others who are overwhelmed at the prospect of looking forward at their careers (1–6). By no means is my scientific journey over, but since 2002, I have been on a journey that I did not realize I was on. Now that the paper introducing the mothur software package is 10 years old and has become the most cited paper published by *Applied and Environmental Microbiology* (7) (Fig. 1), it is worth stepping back and using the continued development of mothur as a story that has parallels to many other research stories.

I fondly recall preparing a poster for the 2002 meeting of research groups supported by the NSF-supported Microbial Observatories Program. I wanted to triumphantly show that I had sequenced more than 600 16S rRNA gene sequences from a single 0.5-g sample of Alaskan soil. This was greater sequencing depth than anyone else had achieved for a single sample. As I was preparing the poster, I walked into the office of Jo Handelsman, my postdoctoral research advisor at the University of Wisconsin, and laid out the outline for the poster. She asked if I could add one of those “curvy things,” a rarefaction curve, to show where I was in sampling the community. Rarefaction curves and attempts to estimate the taxonomic richness of soil had become popular because of the impactful review by Jennifer Hughes and her colleagues (8). Their seminal paper introduced the field to operational taxonomic units (OTUs), rarefaction curves, and richness estimates. I do not recall whether my poster had a rarefaction curve on it, but Jo’s question and that review article primed my career.

## 

### Introducing DOTUR and friends.

When Jo asked me to generate a rarefaction curve for the poster, the request was not trivial. How would I bin the sequences into OTUs? Hughes and her colleagues did it manually and with fewer than 300 sequences. Although I could possibly do that for my 600 sequences, my goal was to generate 1,000 sequences from the sample and to repeat that sampling effort with other samples. I needed something that could be automated. Furthermore, the software that Hughes used to build rarefaction curves, EstimateS (4), required a series of tedious data formatting steps to perform the analyses we were interested in performing. I had found my first problem. How would I assign sequences to OTUs and use that data to estimate the richness and diversity of a sample? The second problem would involve comparing the abundance of OTUs found in one sample to another sample. The solution to the first problem, DOTUR (*d*istance-based *OTU*s and *r*ichness), took us 2 years to develop (9). DOTUR did two things: given a matrix quantifying the genetic distance between pairs of sequences, it would cluster those sequences into OTUs for any distance threshold to define the OTUs and then it would use the frequency of each OTU to calculate a variety of alpha diversity metrics. The solutions to the second problem would come from our work to develop software, including ∫-LIBSHUFF (10), SONS (*s*hared *O*TUs and *s*imilarity) (11), and TreeClimber (12). Around the same time, Catherine Lozupone and Rob Knight were developing their UniFrac tools to compare communities with a phylogenetic rather than OTU-based approach (13, 14). With these tools, the field of microbial ecology had a quantitative toolbox for describing and comparing microbial communities. Along the way Jo and I would demonstrate the utility of such tools for answering questions like how many OTUs were there in that sample of Alaskan soil and how many sequences were needed to sample each of those OTUs (15)? Where were we in the global bacterial census (16)? How does the word usage of *Goodnight*, *Moon* compare to that of *Portrait of a Lady*, and more importantly, how is this relevant to microbial ecology (17)? Most edifying were the more than 2,400 papers that used DOTUR, SONS, TreeClimber, or ∫-LIBSHUFF to facilitate their own research questions (Web of Science, 1 October 2019). Had we waited to solve all of the problems that plagued 16S rRNA gene sequencing, we would still be waiting.

It is important to remember that we knew there were many problems with 16S rRNA gene sequencing. We knew there were biases from extractions and amplification (18–23). We knew there were chimeras (24–27). We knew that bacteria varied in their *rrn* copy number. Generating a distance matrix was a prerequisite to using my tools. This was not trivial, but by cobbling together other tools, it was possible. We would assemble, trim, and correct Sanger sequence reads using Chromas or STADEN (28), align the sequences using ClustalW (29) or ARB (30), check for chimeras using partial treeing or Bellerephon (27), and calculate a pairwise distance matrix using DNADIST from the PHYLIP package (31). At the time, we knew that we had only a loose concept of a species based on these distances (32). We hoped that an OTU defined as a group of sequences more than 97% similar to each other would be a biologically meaningful unit regardless of whether it fit our notion of a bacterial species. At the time, I felt that the biggest problems that I could solve were how to cluster the sequences into OTUs and how to use those clusterings to test our hypotheses. The only tool available at the time that automated the clustering step was FastGroup, which implemented an approximation of the single linkage algorithm (33). The high cost of sequencing was also an impediment to experimentation and analysis in microbial ecology. It was rare for a study design to have experimental replicates so that one could perform a statistical test to compare treatment groups. For example, in our testing, we frequently used a data set comparing Scottish soils from Alison McCaig and colleagues (34). This data set consisted of two experimental groups, each replicated three times with 45 sequences per replicate. Although great focus has been placed on the depth of sampling afforded by 454 and Illumina sequencing, the true benefit of the modern sequencing platforms is the ability to affordably sequence a large number of technical and biological replicates. In my opinion, this expansion in the number of replicates more than makes up for the potential limitations incurred by their shorter read length. In spite of the many technical challenges, we had excuses and heuristics to solve problems that served our needs. It is telling that a recent review of “best practices” in generating and analyzing 16S rRNA gene sequences shows that we still have not solved many of these issues and that we have, of course, identified additional problems (35).

As we developed these tools, I found a unique niche in microbiology. My undergraduate and graduate training as a biological engineer prepared me to think about research questions from a systems perspective, to think quantitatively, and to understand the value of using computer programs to help solve problems. As an undergraduate student, I learned the Pascal programming language and promptly forgot much of it. Although it was a good language for teaching programming concepts, it did not catch on outside the classroom. Later, I learned MATLAB. Because it was an expensive commercial programming environment and never caught on with biologists, I also forgot much of it. Even if I forgot the programming syntax of these languages, what learning these languages taught me was the logic and structure of programming. As a postdoc, I would use this background to learn the Perl programming language to better understand how LIBSHUFF (i.e., LIBrary SHUFFle), a tool for comparing the structure of two communities, worked since it was written in Perl (36). After writing my own version of LIBSHUFF, ∫-LIBSHUFF, and seeing the speed of the version written in C++ by my collaborator, Bret Larget, I converted my Perl version of DOTUR into C++. At the time, the conversion from Perl to C++ seemed like an academic exercise to learn a new language. My Perl version of DOTUR took a minute or so to process the final collection of 1,000 sequences, and the C++ version took seconds. Was that really such a big difference? In hindsight, as we now process data sets with tens of millions of sequences, the decision to learn C++ was critical. The ability to pick up computer languages to solve problems, enabled by my prior training in engineering, was a skill that was virtually unheard of in microbiology. Today, researchers without the ability to program are at a significant disadvantage (37).

### Introducing mothur.

Shortly after DOTUR was published, I received an e-mail from Mitch Sogin, a scientist at the Marine Biology Laboratory (Woods Hole, MA), who asked whether DOTUR could handle more than a million sequences. Without answering his question, I asked where he found a million sequences. Little did I know that his e-mail would represent another pivot in the development of these tools and my career. His group would be the first to use 454 sequencing technology to generate 16S rRNA gene sequences (38). Although DOTUR could assign millions of sequences to OTUs, it was slow and required a significant amount of RAM (random access memory). As I left my postdoc to start my independent career across the state from Sogin’s lab at the University of Massachusetts in Amherst, my plan was to rewrite DOTUR, SONS, ∫-LIBSHUFF, and TreeClimber for the new world of massively parallelized sequencing. The new tool would become mothur.

Milling about at a poster session at the 2007 ASM General Meeting in Toronto, Canada, I ran into Mitch who asked what my plans were for my new lab. I told him that I wanted to make a tool like ARB, a powerful database tool and phylogenetics package (30), but for microbial ecology analysis. His retort was, “You and what army?” Up to that point, I had written every line of code and had been answering many e-mails from people asking for help. He was right, I would need an army. It would be difficult, but I needed to learn to let go and share the development process with someone else. My “army” ended up being Sarah Westcott, who has worked on the mothur project from its inception. Today, mothur is nearly 200,000 lines of code, and Sarah has touched or written nearly every line of it. Beyond writing and testing mothur’s code base, she has become a conduit for many who are trying to learn the tools of microbial ecology. She patiently answers questions via e-mail and on the package’s discussion forum (https://forum.mothur.org). The community and I are lucky that Sarah has stayed with the project for more than a decade. To be honest, such dependency on a single person makes the project brittle. In hindsight, it would have been better to have developed mothur with more of an “army” or team so that there is overlap in people’s understanding of how mothur works. Although a distributed team approach might work in a software engineering firm, it is not practical in most academic environments where there is limited funding. There are certainly projects that make this work, but they are rare.

### Competition has been good and healthy.

mothur has not been developed in a vacuum, and it does not have a monopoly within the field. As indicated above, each of our decisions was made in the historical context of the field and with constant pressure from others developing their own tools for analyzing 16S rRNA gene sequence data. Competition has been good for mothur and for the field.

From the beginning, there have been online tools available at the Ribosomal Database Project (RDP) (39), greengenes (40), and SILVA (41). These tools allowed users a straightforward method of comparing their data to those collected in a database. There are two primary downsides to these tools. First, researchers running the online tool must pay the computational expenses. When their hardware becomes outdated because it is expensive to replace or maintain, processing times slow down. Eventually, this limitation would result in the termination of the greengenes website. Second, these platforms provide a one-size-fits-all analysis. These tools allow a user to analyze only 16S rRNA gene sequences and in some cases 18S rRNA gene sequences. If a user sequences a different gene, then the tool will not serve them. These observations resulted in two design goals we have had with mothur: bringing the analysis to a user’s computer and separating a tool from a specific database. For example, we commonly use a sequence alignment method that was originally developed for greengenes (42), but we use a SILVA-based reference alignment because of its superior quality (43, 44). In addition, we offer the Bayesian classifier developed by the RDP (45) and allow users to train it to any database they want, including customized databases. In both examples, users can align or classify non-rRNA gene sequence data. As the bioinformatics tools have matured, both RDP and SILVA now offer integrated pipelines for analyzing large data sets, albeit in one-size-fits-all black box implementations.

With the growth in popularity of 16S rRNA gene sequencing, there has naturally been an expansion in the number of people developing tools to analyze these data. Months after the paper describing mothur was published, the paper describing QIIME was published (46). Over the past 10 years, many have attempted to create analogies comparing the two programs: Pepsi versus Coke, Apple versus Windows, etc. It is never clear which software is which brand and whether the comparisons are meant as a complement or an insult. Regardless, both programs are very popular. From my perspective, most of the differences are cosmetic (http://blog.mothur.org/2016/01/12/mothur-and-qiime/). To me the most meaningful difference between mothur and QIIME is the choice of algorithms used to cluster sequences into OTUs. QIIME’s advocacy for open and closed reference clustering and USEARCH- or VSEARCH-based *de novo* clustering results in lower-quality OTU assignments relative to the *de novo* clustering algorithms available within mothur (47, 48). QIIME is set of wrapper scripts that help users to transition data between independent packages. For example, with QIIME (through version 1.9.1), it was even possible to run mothur through QIIME. One can also run the Bayesian classifier through QIIME using the original code developed by the RDP. Structuring QIIME as a set of wrappers caused great frustration for many users because there were numerous software dependencies that had to be installed. The benefits included the ability for users to access a wider set of tools and for developers to tie their tool into the popular software package. Although the QIIME developers would go on to create virtual machines and use packaging tools to simplify installation, these fixes required sophistication by users who we knew struggled with the basics of navigating a command line. In contrast, when a user runs mothur, they are running mothur. The Bayesian classifier code that is in mothur is a rewritten version of the original code. When we rewrite someone’s software, we do it with an eye to improving performance, access, and utility for non-16S rRNA gene sequence data. For example, while 454 data were popular, PyroNoise was an effective tool for denoising flowgram data (49). Running the original code required a large Linux computer cluster and knowledge of bash and Perl scripting. When we rewrote the code for mothur, we made it accessible to people using any operating system with a simple command interface (i.e., trim.flows and shhh.flows). Our approach requires significant developer effort but saves considerable user effort. As this benefit is multiplied across thousands of projects, the savings to users has been considerable.

Beyond the large packages like mothur and QIIME, there has been significant growth in stand-alone software tools for sequence curation (e.g., PyroNoise [49], PANDAseq [50], and DADA2 [51]), chimera checking (e.g., UCHIME [52], ChimeraSlayer [53], and Perseus [54]), and clustering (e.g., USEARCH [55], VSEARCH [56], and Swarm [57]). Where possible and when warranted, we have implemented many of these algorithms directly into mothur. We have also used this diversity of methods to perform head-to-head comparisons. Most notable is the area of clustering algorithms where there have been a large number of algorithms developed without an obvious method to objectively compare them (47, 48, 58, 59). We applied an objective metric, Matthew’s correlation coefficient (MCC), to evaluate numerous algorithms for clustering sequences into OTUs. By performing this type of analysis, we were able to objectively compare the algorithms, make recommendations to the field, and develop new algorithms that outperformed the existing ones. Beyond evaluating clustering algorithms, we have also evaluated methods of denoising sequence data (60–62), assessed reference alignments (43, 44), considered the importance of incorporating secondary structure information in alignments (63), quantified the variation along the 16S rRNA gene (44), and compared the statistical hypotheses tested by commonly used tools (64). We have embraced the competition and diversity of all methods being used to analyze amplicon data. This competition forces us to identify the strengths and weaknesses of various methods so that we can make recommendations to other researchers.

### mothur’s core principles.

As mothur has evolved with the needs of the community, several core principles have emerged that direct its development. First, mothur is a free, open-source software package. This has been critical in shaping the direction of mothur. We were content for mothur to be an improved combination of DOTUR and SONS and leverage existing tools for other steps. Yet, we quickly appreciated the need for providing other steps in a sequence analysis pipeline to make other tools more accessible. This decision was motivated by learning that the code for greengenes’s (42) and ARB/SILVA’s aligners were not open source or publicly available. Thus, we realized that such an important functionality needed to be open to the community (43). More recently, the rejection of closed-source, commercial tools can be seen by the broader adoption of open-source alternatives. This has been the case with the rising popularity of VSEARCH over USEARCH within the microbial ecology community (55, 56).

**FIG 2:**
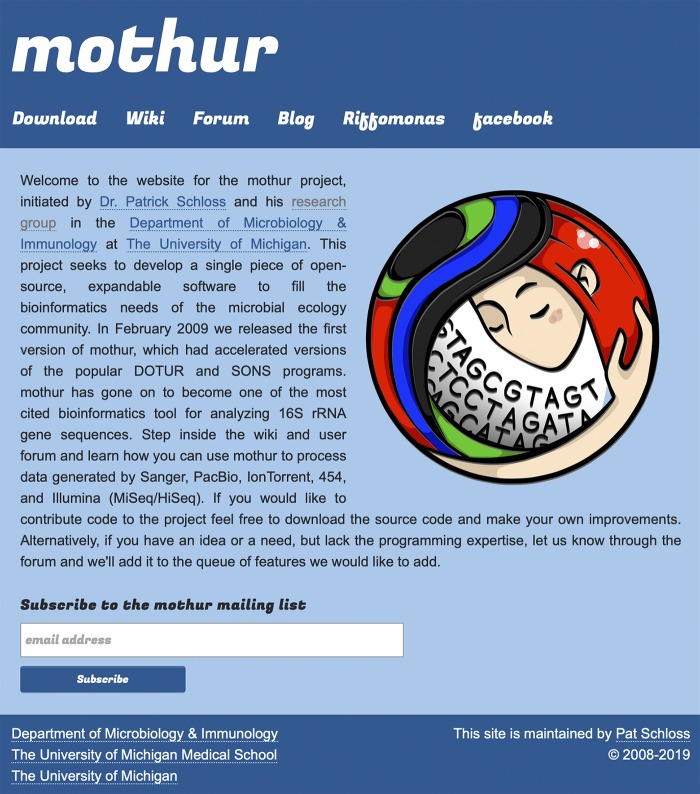
mothur homepage. From the mothur home page at www.mothur.org, users can download mothur, access a user forum, navigate a wiki with extensive documentation, find blog posts that provide additional examples of how to use mothur, join the mothur Facebook group, and subscribe to the mothur mailing list.

Related to ensuring that mothur’s code is open source, our second core principle is that we maintain transparency to our users. Perhaps a user does not need to interrogate every line of code, but they need to understand what is happening. Many programs, including online workflows, encapsulate large elements of a pipeline in a single command. In contrast, mothur forces the user to specify each step of the pipeline. Although the former approach makes an analysis easier for a beginner, it stifles users that need greater control or understanding of the assumptions at each step. This control over the pipeline has made it easier for researchers to customize databases or adapt the pipeline to analyze non-16S rRNA gene sequence data. Furthermore, we have provided ample instructional materials to teach users how to implement robust pipelines and the theory behind each step through the project’s website (https://www.mothur.org) (Fig. 2).

Third, as I mentioned above, there has been a plethora of methods proposed for generating amplicon sequence data and curating, aligning, checking for chimeras, classifying, and clustering the data. I am proud of the data-driven approach we have taken to comparing these methods. A description of a new method has limited value if it is not benchmarked against other methods or control data sets. Through this core principle and mothur’s large reach into the community, we have helped to develop standards in the analysis of 16S rRNA gene sequence data.

Fourth, a focus on enabling reproducibility has always been central to the functionality of mothur. From the beginning, mothur’s logfiles have represented a transcript of the user’s command and outputs. When it became clear that researchers were not submitting their sequence data to the Sequence Read Archive (SRA), we worked with the SRA developers to create a mothur command (make.sra) that creates a package for submitting sequence data through a special mothur portal. A more ambitious project had its seed on 1 April 2013 when we announced a new “function” in mothur: write.paper. The new command required that the user provide a 454 sff file and a journal title or impact factor. With this information, mothur would generate a paper. This April Fools’ Day joke was poking fun at software that provided an analysis black box but also at many users’ sentiments that data analysis should be so cut and dry. A few years later, we revisited this concept in the scope of reproducibility. Why not explicitly script an analysis from downloading data from the SRA through the rendering of a paper ready for submission? This idea gave rise to the development of the Riffomonas reproducible research tutorial series that enables researchers to write their own version of write.paper (65).

Perhaps the most important core principle is that my research group uses mothur to analyze the data we generate. This has proven critical, as it again represents transparency and hopefully provides confidence to mothur’s users that we are not making recommendations that we do not follow ourselves.

### Challenges of making open source count.

Anyone can post code to GitHub with a permissive license and claim to be an open-source software developer. Far more challenging is engaging the target community to make contributions to that code. Frankly, we have struggled to expand the number of people who make contributions to the mothur code base. One challenge we face is that if we looked to others to contribute code to mothur, they would need to know C++. Given the paucity of microbiologists that can program in a compiled language like C++, expecting that community to provide contributors who can write code in a syntax that prizes execution efficiency over developer efficiency was not realistic. In contrast, the QIIME development team could be more distributed because their code base was primarily written in Python, which prizes developer efficiency over execution efficiency. QIIME is a series of wrappers that allow users to execute other developers’ code, making the use of a scripting language like Python attractive. Their choices resulted in many tradeoffs that have impacted ease of installation, usability, execution speed, and flexibility. If we were offered funding to rewrite mothur, we would likely rewrite it as an R package that leaned heavily on the R language’s C++ interface packages. Of course, such choices are always best in hindsight. When we started developing mothur, the ability to interface between scripting languages like R and Python and C++ code was not as well developed as it is today. For example, the modern version of the Rcpp package was first released in 2009 and its popularity was not immediate (66). The development of mothur has been a product of the environment that it was created in. Although these decisions have largely had positive outcomes, there have been tradeoffs that caused us to sacrifice other goals.

Beyond contributing to the mothur code base, we sought out other ways to include the community as developers. The paper describing mothur included 15 coauthors, most of whom responded to a call to provide a wiki page that described how they used an early version of mothur to analyze a data set. Our vision was that authors might use the mothur wiki to document reproducible workflows for papers using mothur but also to provide instructional materials for others seeking to adapt mothur for their uses (https://www.mothur.org/wiki). Unfortunately, once the incentive of coauthorship was removed, researchers stopped contributing their workflows to the wiki. Again, this vision and the lack of the community’s adoption of wikis as a mechanism for reporting workflows were products of the environment. Although wikis were popular in the late 2000s, they lacked the ability to directly execute the commands that researchers reported. Such technology would not be possible until the creation of IPython notebooks (2011) and R markdown (2012). Another problem with the wiki approach was that potential contributors did not see the wiki as a community resource. I frequently received e-mails from scientists telling me that there was a typo on a specific page when the intention was that they could correct the typos without my input. We have been more successful in soliciting input and contributions from the user community through the mothur discussion forum and GitHub-based issue tracker. As mothur has matured, we have been dependent on the user community to use these resources to tell us what features they would like to see included in mothur and where the documentation is confusing or incomplete (https://forum.mothur.org). Often we can count on people not directly affiliated with mothur to provide instruction and their own experience to other users on the forum. We are constantly trying to recruit our “army” and are happy to take any contributions we can. Whether the contributions are to the code base, discussion forum, or suggestions for new tools, these contributions have been invaluable to the growth and popularity of mothur.

### Failed experiments.

If we never failed, we would not be trying hard enough. Over the past decade, we have tried a number of experiments to improve the usability and utility of mothur.

One of our first experiments was to use mothur to generate standard vector graphic (SVG)-formatted files of heatmaps and Venn diagrams depicting the overlap between microbial communities. Such visuals were helpful for exploring data; however, I quickly realized that I would never put a mothur-generated figure into a paper I wrote. Such visuals require far too much customization to be publication quality. Although QIIME has incorporated visualization tools through the Emperor package (67), the challenge of users taking default values has downsides, especially when those defaults do not follow good data visualization principles. For example, ordinations with black backgrounds and three-dimensional (3-D) ordinations in a two-dimensional (2-D) medium now litter the literature. Instead, we have encouraged users to use R packages to visualize mothur-generated results using the minimalR instructional materials that I have developed (http://www.riffomonas.org/minimalR/).

**FIG 3:**
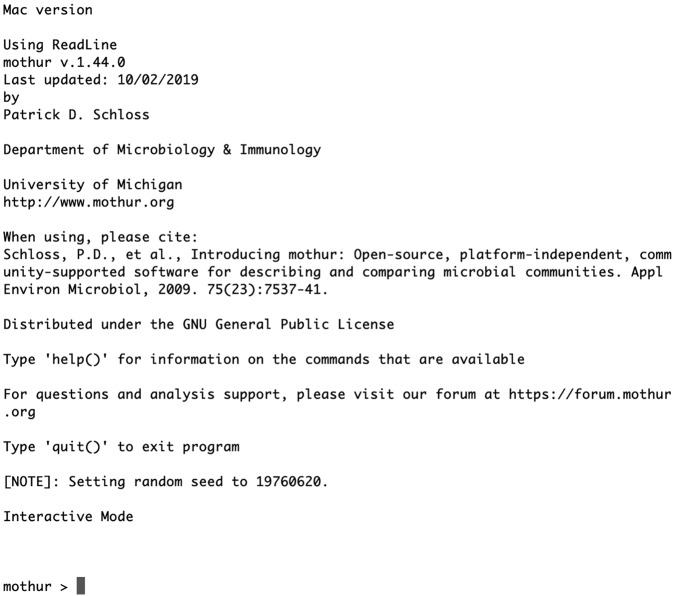
Start-up window when running mothur in Mac OS X in the interactive mode. mothur can also be run on Windows or Linux. In the interactive mode, users enter individual commands at the mothur prompt. Alternatively, users may run mothur by supplying commands from the command line or using batch scripts.

A second experiment was the creation of a graphical user interface (GUI) for running mothur. Forcing users to interact with mothur through the command line has been a significant hurdle for many (Fig. 3). Unfortunately, the development effort required to create and maintain a GUI is significant and there is limited funding for such efforts. The newest version of QIIME (starting with version 2.0.0) has emphasized interaction with the tools through a GUI (68), and the related QIITA project offers a web-based GUI (69). It remains to be seen how this experiment will go. Another downside of using a GUI is that there is a risk that reproducibility will suffer if users do not have a mechanism to document their mouse clicks. A significant downside for web interfaces is the frequent inability to document or return to old versions of software and databases. As was experienced with greengenes, if the website is terminated, reproducing old analyses becomes impossible. In mothur, documentation of commands and parameter values is explicit in that users can provide a file with a list of commands and the software returns a logfile with all commands and output recorded. Given the heightened focus on reproducibility in recent years, we have extended significant effort in developing instructional materials teaching users how to organize, document, and execute reproducible pipelines that allow a user to go from raw sequence data to a compiled paper (65, 70).

A final example of a failed experiment was a collaboration with programmers through Google Summer of Code to develop commands in mothur that ran the random forest and SVM (support vector machine) machine learning algorithms. Similar to the challenges of developing attractive visuals, fitting the algorithms’ hyperparameters, testing, and deploying the resulting models require a significant amount of customization. Furthermore, machine learning is an active area of research where methods are still being developed and improved. Thankfully, there are numerous R and Python packages that do a better job of developing these models (71, 72).

In each of our “failed” experiments, the real problems were straying from what mothur does well and failing to grasp what we really wanted the innovation to do.

### The future.

I will continue to develop mothur for as long as other researchers find it useful. One challenge of such a plan is maintaining the funding to support its development. The development of mothur was initially enabled by a subcontract from a Sloan Foundation grant to Mitch Sogin to support his VAMPS (Visualization and Analysis of Microbial Population Structures) initiative. We used that seed funding to secure an NSF grant and then a grant from NIH for tool development as part of their Human Microbiome Project. Since that project expired in 2013, we have not had funding to specifically support mothur’s development. I have been fortunate to have start-up and discretionary funds generated from other projects to help support mothur. Although there is funding for new tools, there appears to be little appetite by funders to support existing tools. Emblematic of this was the NIH program, Big Data To Knowledge (BD2K), which solicited proposals through the program announcement “Extended Development, Hardening and Dissemination of Technologies in Biomedical Computing, Informatics and Big Data Science (PA-14-156).” This opportunity appeared perfect, except that the National Institute of Allergy and Infectious Diseases (NIAID), the primary supporter of microbiome research at NIH, did not participate in the announcement. Tools like mothur are clearly successful, but they need funding mechanisms to continue to mature and support the needs of the research community.

As with anything in science, methods become passé. When we first developed mothur, T-RFLP (terminal restriction fragment length polymorphism) and DGGE (denaturing gradient gel electrophoresis) were still commonly used. Today it would be hard to argue that data from those methods meaningfully advance a study relative to what one could obtain using 16S rRNA gene sequence data. Looking forward, many want to claim that amplicon sequencing is today’s DGGE. They claim that researchers should instead move on to shotgun metagenomic sequencing. It is important to note that the two methods answer fundamentally different questions. 16S rRNA gene sequence data describes the taxonomic composition, while metagenomic sequence data tells a researcher about the functional potential and genetic diversity of a community. Both tools provide important information, but they cannot easily replace each other. Although metagenomic data does provide highly resolved taxonomic information, its practical limit of detection is at least an order of magnitude higher than that of amplicon data. For example, we analyzed 10,000 16S rRNA sequences from each of about 500 subjects (73). We can think of this as representing about 1,000,000 genome equivalents (10,000 16S rRNA genes/subject × 500 subjects/5 16S rRNA gene sequences/genome). Assuming a genome is 4 Mbp, this would represent a sequencing depth of 4 Tbp. Although such a sequencing effort is technically possible, the cost of such an endeavor would be considerable and unlikely to be pursued by most researchers. We estimate that generating and sequencing the libraries at the University of Michigan sequencing core would cost approximately $150 per library. The parallel 16S rRNA gene sequences data would cost approximately $8 per library. Furthermore, analyzing such a large data set with an approach that captures the full genetic diversity of the community would be financially and technically prohibitive. Going forward, sequencing technologies will continue to evolve to capture longer and more high-quality data, and there will always be a need for characterizing the taxonomic diversity of microbial communities. With this in mind, there will always be a place for tools like mothur that can analyze amplicon sequence data.

Of course, this does not mean that such tools will remain static. We see three key areas that we will continue to help the field to move forward. First, just as we adapted through the transitions from Sanger to 454 to MiSeq and PacBio sequencing platforms (60–62), we must learn whether data from Oxford Nanopore and other developing sequencing technologies can be an alternative sequencing approach that generates sequence data that is the same quality as existing approaches; thus far, the approach has significant shortcomings for sequencing 16S rRNA gene sequences (74). As with the earlier platforms, we must better understand its error profile so that sequencing errors can be corrected. We have learned that moving forward requires that we maintain or improve sequence quality. No doubt, data sets and read lengths will improve, but these advances should not be made at the cost of data quality. Second, with these improvements, we will need to continue to improve our algorithms. We have already seen that attempts to use low-quality MiSeq and HiSeq data caused computational problems leading to the creation of open and closed reference clustering methods, which attempted to circumvent those problems (75, 76). Unfortunately, comparative analyses showed that these methods fail relative to *de novo* clustering methods (47, 48). More work is needed to improve reference-based clustering methods so that larger data sets can be analyzed without sacrificing the quality of OTU assignments. Finally, there are ongoing controversies that need further exploration. These controversies include the validity and utility of amplicon sequence variants (77), the wisdom of removing low-frequency sequences (78), and methods of identifying and removing contaminant 16S rRNA gene sequences (79, 80). With each of these areas of development, the broader community can count on our same data-driven approach to answer these questions. It is common for researchers to comment that they pick a specific method or deviate from a suggestion because they “like how the data look.” When pressed for an objective definition of how they know the data look “right,” they go quiet. Through the use of data where we actually know what looks right and objective metrics of quality, we will continue to base recommendations on data rather than a gut feeling.

### Conclusion.

In the paper announcing mothur, we commented that the relationship between 16S rRNA gene sequencing and analysis is very much like the Red Queen in Lewis Carroll’s book, *Through the Looking-Glass*. Although some disagreed with this analogy (81), I still feel it is apt. The sequencing technology and rapacious appetite of researchers continue to race on. At the same time, bioinformatics tools must adapt to facilitate our research. I am confident that mothur will be up to this exciting challenge. Beyond its utility for analyzing amplicon sequence data, mothur’s history provides lessons that are helpful for other projects that hope to develop a long historical arc. First, mothur is a product of its time. We have always sought to solve a current need to the best of our ability with the tools we had at the time. There are certainly caveats to any analysis of 16S rRNA gene sequence data, but if we had waited until those caveats were resolved, the field would never have progressed. Similarly, we made design choices that we probably would not have made had we started the project today. Second, as we have developed mothur, we have attempted to do so in a data-driven approach where we compare multiple methods. It has not merely been enough to propose a new method: we must show that it meaningfully advances the field. Third, through our failures and successes, we have learned to focus on what mothur is good at and create products separate from mothur when distinct needs arise. For example, we have learned that mothur should not have a graphical interface or data visualization tool. Instead, we provide instructional materials to teach users how to use the command line interface and other computational skills like programming in R for data visualization. Finally, mothur was born out of a need for automating the analysis of large 16S rRNA gene sequence data sets. It has been refreshing to see the computational skills of the microbial ecology field grow over the past 2 decades. Looking ahead, we must all take stock of the challenges we face in microbial ecology and how our individual skills and interests can address these challenges to turn them into opportunities.
